# Validated assays for the quantification of C9orf72 human pathology

**DOI:** 10.1038/s41598-023-50667-3

**Published:** 2024-01-08

**Authors:** S. E. Salomonsson, A. M. Maltos, K. Gill, O. Aladesuyi Arogundade, K. A. Brown, A. Sachdev, M. Sckaff, K. J. K. Lam, I. J. Fisher, R. S. Chouhan, V. S. Van Laar, C. B. Marley, I. McLaughlin, K. S. Bankiewicz, Y.-C. Tsai, B. R. Conklin, C. D. Clelland

**Affiliations:** 1 Weill Institute for Neurosciences, University of California San Francisco, San Francisco, CA USA; 2 Memory & Aging Center, Department of Neurology, University of California San Francisco, San Francisco, CA USA; 3 Gladstone Institutes, San Francisco, CA USA; 4 Department of Neurological Surgery, The Ohio State University, Columbus, OH USA; 5 The Gene Therapy Institute, The Ohio State University, Columbus, OH USA; 6 Pacific Biosciences, Menlo Park, CA USA; 7 Departments of Medicine, Ophthalmology, and Pharmacology, University of California San Francisco, San Francisco, CA USA

**Keywords:** Genetics, Gene expression, Gene regulation, Mutation, Sequencing, Neurology, Neurological disorders, Molecular medicine

## Abstract

A repeat expansion mutation in the *C9orf72* gene is the leading known genetic cause of FTD and ALS. The *C9orf72-*ALS/FTD field has been plagued by a lack of reliable tools to monitor this genomic locus and its RNA and protein products. We have validated assays that quantify *C9orf72* pathobiology at the DNA, RNA and protein levels using knock-out human iPSC lines as controls. Here we show that single-molecule sequencing can accurately measure the repeat expansion and faithfully report on changes to the *C9orf72* locus in what has been a traditionally hard to sequence genomic region. This is of particular value to sizing and phasing the repeat expansion and determining changes to the gene locus after gene editing. We developed ddPCR assays to quantify two major C9orf72 transcript variants, which we validated by selective excision of their distinct transcriptional start sites. Using validated knock-out human iPSC lines, we validated 4 commercially available antibodies (of 9 tested) that were specific for C9orf72 protein quantification by Western blot, but none were specific for immunocytochemistry. We tested 15 combinations of antibodies against dipeptide repeat proteins (DPRs) across 66 concentrations using MSD immunoassay, and found two (against poly-GA and poly-GP) that yielded a 1.5-fold or greater signal increase in patient iPSC-motor neurons compared to knock-out control, and validated them in human postmortem and transgenic mouse brain tissue. Our validated DNA, RNA and protein assays are applicable to discovery research as well as clinical trials.

## Introduction

Heterozygous expansion of an intronic GGGGCC repeat in the *C9orf72* (C9) gene is the most frequent known genetic cause of both frontotemporal dementia (FTD) and amyotrophic lateral sclerosis (ALS)^1–3^. C9-ALS/FTD pathology is thought to result from toxic products derived from expression of the *C9orf72* repeat expansion itself. For instance, transcripts that harbor a large repeat expansion produce toxic dipeptide repeat proteins (DPRs) through repeat-associated non-AUG (RAN) translation^4–13^.

Tools to accurately measure gene products from both the non-expanded and expanded gene are critical to understanding *C9orf72* gene function in non-diseased and diseased states. We were inspired by another published effort to validate C9orf72 antibodies for Western blot^14^, which has been as a touchstone by many in the field, to publish our efforts to validate DNA, RNA and DPR reagents and methods using engineered patient and wild-type (WT) knock-out (KO) iPSC lines. Here we report on reagents and methods (such as single molecule sequencing to size the repeat expansion, or using KO iPSC lines for RNA and protein reagent validation) that greatly improve the characterization of *C9orf72* and its gene products.

## Results

### Single-molecule sequencing distinguishes repeat expansion lengths in patient-derived iPSC lines

**Figure 1:**
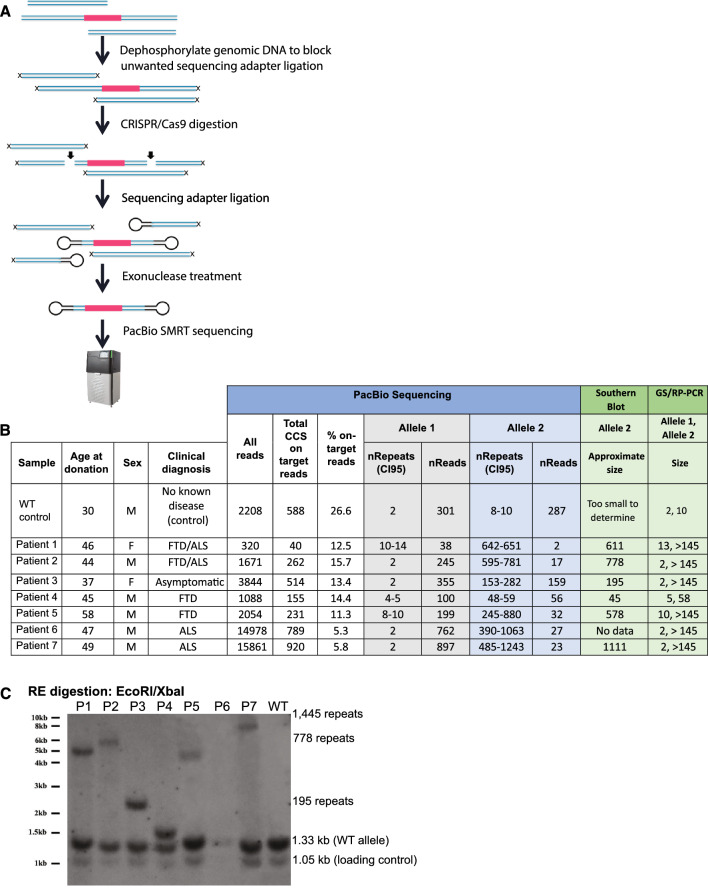
Pacific Biosciences (PacBio) single-molecule sequencing to determine the *C9orf72* repeat size in 8 iPSC lines. (**A**) Schematic of the pipeline used to generate the library for single-molecule sequencing. We excise the repeat region (red) from high-molecular-weight DNA using CRISPR and guide RNAs flanking the repeat regions (arrows). We then seal the CRISPR-generated double-strand breaks by ligating in sequencing adapters. Subsequent exonuclease treatment results in an enrichment for the excised repeat region, which is sealed at both ends. The enriched repeat regions are then subjected to PacBio SMRT sequencing. Because the sequenced molecules are circular, the sequencing reaction can read through them more than once, which increases the accuracy of the sequencing data. Barcoding allows us to multiplex samples to reduce sequencing costs. (**B**) We sequenced 3–5 µg of DNA from 1 WT-control iPSC line and 7 iPSC lines from patients harboring expansions of the *C9orf72* repeat. Allele-specific SNPs allowed us to distinguish the repeat regions from the two *C9orf72* alleles (Allele 1 and Allele 2) in each cell line. On-target reads are reads that sequenced the entire excised region (including the repeat region and flanking DNA) 3 times or more (> 3 pass criteria). Within these reads, we counted the number of GGCCCC repeats starting right after an anchor (CGCCC) 5′ to the repeat region. Repeat lengths and associated read counts are reported for each allele of each cell line and compared to repeat length estimated by Southern blot and GS/RP-PCR. Repeat lengths estimated by Southern blot were comparable to mean repeat lengths determined by single-molecule PacBio sequencing, while GS/RP-PCR could not determine repeat lengths > 145. (**C**) Southern blot of nuclear DNA from WT-control and patient iPSCs listed in (**B**). After EcoR/XbaI digestion, a loading control fragment (1.05 kb), WT fragment (1.33 kb) and fragments with repeat expansions of various lengths were detected. Southern blot required 20 µg of input DNA (vs. 3–5 µg input for PacBio sequencing) and a sample with 14 µg (P6) failed detection, demonstrating the insensitivity of Southern blot.

**Figure 2:**
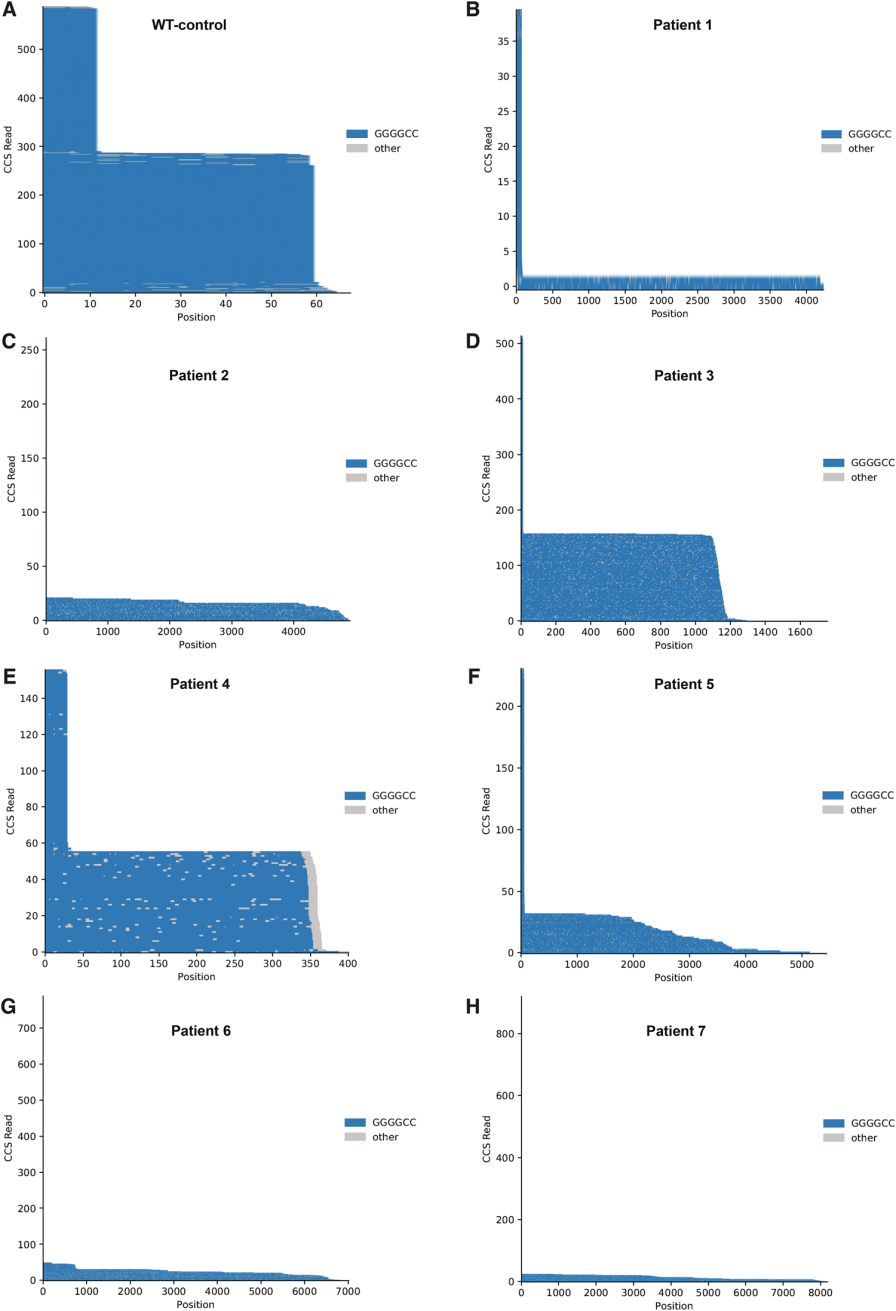
Pacific Biosciences (PacBio) single-molecule sequencing traces of the *C9orf72* repeat region for patient and control cell lines. (**A**–**H**) Sequencing traces showing the number of circular consensus sequencing (CCS) reads per repeat count. In the sequencing traces, each horizontal line represents one sequenced molecule of DNA. Blue color depicts on-target sequencing with GGGGCC repeat, grey color depicts sequencing error. Each molecule is anchored to an adjacent, non-repeat region (CGCCC) which is not included In the total repeat count. Y-axis = CCS count. In the WT line, repeat length has a bimodal distribution with roughly equal numbers of reads containing 2 or 10 repeats, indicating that one allele has 2 repeats and the other 10. In the patient lines, a bimodal distribution is present but not always as apparent: all lines show a peak with a low repeat number (2–10), corresponding to the unexpanded allele, and the expanded allele size can vary across cell lines from different donors.

Currently, long-range PCR^15^ and Southern blot^16^ are used to clinically diagnose repeat expansion mutations in the *C9orf72* gene, but these approaches lack precision, especially for large expansions. Sizing repeat expansions above ~ 100 repeats is not possible using traditional sequencing techniques that require amplification, because amplification fails across GC-rich repetitive DNA regions. Patients can have *C9orf72* repeats into the thousands! Newer optical genomic mapping techniques have been reported to size large repetitive regions^17^ (even up to 45 kb) but they have limited resolution, which could result in missing as many 80 hexanucleotide C9-repeats. We turned instead to single-molecule sequencing, which has been demonstrated to traverse the expanded repeats of *C9orf72* in plasmid^18^ and human tissue^19,20^. We collected patient iPSC lines from previously published or publicly available sources^21–24^ and optimized PacBio single-molecule sequencing of DNA from these lines to size their repeat expansions (Fig. 1). Using Cas9 to generate double-stranded breaks (DSBs) in purified DNA, adapter ligation to capture the cut genomic region of interest, and exonuclease digestion to eliminate DNA fragments without adapters, we were able to enrich for a 3.6–10 kb genomic region centering on the repeat expansion without amplification (Fig. 1A). Sequencing these single, circular DNA fragments gave us a more precise count of the number of repeats present in each cell line (Figs. 1B, 2) than Southern blots (Fig. 1B,C) or gene specific, repeat primed (GS/RP)-PCR (Figs. 1B, S1) did. Single molecule sequencing was more sensitive, requiring an input of only 3 µg DNA versus 20 µg for Southern blot, and more accurate than GS/RP-PCR, which could not quantify repeat lengths greater than 145 repeats.

### Single-molecule sequencing affords better resolution of editing outcomes after dual gRNA excisions of the repeat region than Sanger sequencing does

**Figure 3:**
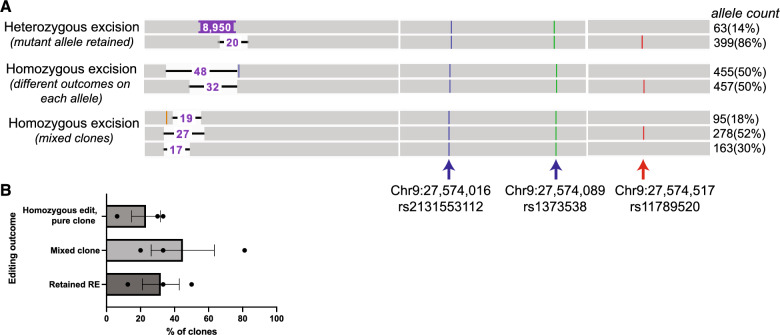
Single molecule sequencing can detect mixed and unedited iPSC clones. (**A**) Examples of editing outcomes viewed in Integrative Genomics Viewer after sorting and cloning single cells from a pool of edited cells. Each horizontal grey bar represents a single sequenced molecule. Alleles can be distinguished by the phased (red arrow) SNP on the WT allele, but not by homozygous SNPs (blue and green arrow) that differ from the reference genome but are shared by both alleles. SNPs are identified by their unique chromosomal position in GRCh38 and reference SNP cluster number (rsID). We attempted to remove the repeat expansion with CRISPR editing. One clone shows a heterozygous excision with a deletion of 20 nucleotides (NTs) on the WT allele and retention of the *C9orf72* repeat expansion (repeats can be variable; 8950 nucleotides or 1491 repeats shown) on the mutant allele. Another clone harbors a homozygous excision with equal read counts across two editing outcomes (48 NT excision on the mutant allele, 32 NT excision on the WT allele). An impure clone shows multiple editing outcomes: a 27 NT excision of the WT allele (52% of sequencing reads), and a 19 NT or 17 NT excision from the mutant allele. The 17 NT excision is twice as abundant as the 19 NT excision in this pool, indicating it is the dominant clone. (**B**) Dual gRNA excision of the repeat expansion across two cell lines with ~ 200 and ~ 1400 repeats in three independent experiments show that editing outcome detected by single-molecule sequencing of clones detects retained repeat expansions (RE) and mixed clones at a high frequency. Error bar = SEM.

We made an isogenic correction of the *C9orf72* repeat expansion by dual-gRNA excision of the repeat region in a patient iPSC line harboring 1400 repeats. Single-molecule sequencing was critical to phasing the repeat expansion and determining the outcomes of edits targeting the expanded allele (Fig. 3). Using Sanger sequencing, it is not possible to determine whether an edit removed the repeat region from both the expanded and WT alleles or just from the WT allele, since the mutant allele fails amplification, and hence detection. Because of its low sensitivity, Southern blot may not reveal the impurity of a cell line harboring a mix of mutant and edited clones. With its high sensitivity and ability to read through long repeat expansions, we found single-molecule sequencing to be superior to current ways of determining editing outcome at the *C9orf72* locus, and recommend it becomes the new gold standard for verifying lines with *C9orf72* edits. Single molecule sequencing can also determine editing outcomes that differ on each allele (Fig. 3A). We also found that 20–80% of our edited clones remained impure even after single-cell sorting (Fig. 3B), indicating that even best practices for line engineering need to be confirmed. These events are impossible to detect with traditional sequencing, which does not differentiate individual alleles, and are likely to be missed by Southern blotting given its requirement for high DNA input.

### ddPCR probes identify and quantify novel *C9orf72* mRNA variant in iPSC-derived neurons

**Figure 4:**
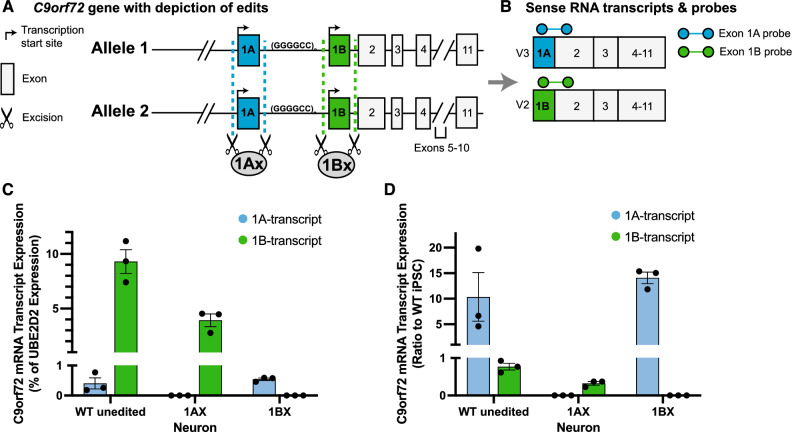
Knock-out validated ddPCR probes to measure 1A- and 1B-containing *C9orf72* mRNA. (**A**) We generated a selective excision of either exon 1A and exon 1B on both alleles to measure the specificity of exon-spanning ddPCR probes (from Table S1). We could not measure variant 1 mRNA given low abundance of this transcript in iPSC derived neurons. (**B**) Probes spanning exon 1A-2 (blue) and 1B-2 (green) measured 1A-containing mRNA (variant 3, V3) and 1B-containing mRNA (variant 2, V2). (**C**) We quantified mRNA in 2-week-old neurons from our WT, exon 1A-excised (1Ax) and exon-1B excised (1Bx) clonal cell lines. Exon 1A-2 probe is specific for 1A-transcripts and exon 1B-2 probe is specific for 1B-transcripts. Most of the transcripts in the cell derive from exon 1B. (**D**) We calculated the transcript expression change for motor neurons derived from each line compared to WT iPSC levels. Although exon 1B-transcripts are the most abundant, levels are equivalent between WT iPSCs and motor neurons (Dunnett’s multiple comparisons test, p = 0.13). However, 1A transcripts increased 10–15 fold compared to iPSCs (Dunnett’s multiple comparisons test, p = 0 < 0.01 for WT neurons and p < 0.001 for 1Bx neurons). Dots = biological replicates. Error bar = SEM.

The *C9orf72* locus is known to produce at least three sense mRNAs: variant 1 (exon 1A-short through exon 5), variant 2 (exon 1B-exon 11) and variant 3 (exon 1A-long through exon 11)^1^. We designed ddPCR probes to span the exon 1A-exon 2 or exon 1B-exon 2 splice junctions to differentiate between the two major splice isoforms, variant 2 and variant 3 (Fig. 4A, Table S1). We were not able to detect variant 1 in our lines, consistent with its low to undetectable expression in human tissue^25,26^.

To validate the specificity of the probes, we made selective and homozygous knock-outs of exon 1A (Fig. 4A, 1Ax) or exon 1B (Fig. 4A, 1Bx) in a non-diseased (WT) control line. Before carrying out our edits, we had engineered our iPSC lines to contain the hNIL transgene cassette with a TET-on system in the CLYBL safe-harbor locus^27,28^. When exposed to doxycycline for three days, the iPSCs harboring the hNIL cassette turn on 3 human transcription factors; NGN2, ISL1, and LHX3, that drive them to differentiate into lower motor neurons (iPSC-MNs). The neurons express high levels of motor neuron markers HB9 and ChAT compared to iPSCs at the time point investigated in our expression studies (2 weeks of age)^27,29^.

We evaluated the performance of each of the probes (Fig. 4B) on *C9orf72* RNA expression in motor neurons derived from the edited and unedited iPSC lines. Most (96%) of the *C9orf72* neuronal mRNAs contained exon 1B, while exon 1A-containing transcripts represented only a small proportion (4%) of total transcripts (Fig. 4C). Homozygous excision of exon 1A eliminated all 1A-containing transcripts (Fig. 4C). Homozygous excision of exon 1B eliminated all 1B-containing transcripts (Fig. 4C). These findings demonstrate that the probes faithfully report on their targeted transcript variant (i.e., demonstrate specificity). Interestingly, we found that excision of exon 1A decreased the amount of 1B transcript by approximately 50%, but excision of exon 1B did not significantly alter the amount of 1A transcript (Fig. 4C). In addition, although 1A-transcripts are a minority, they increased 10–15 fold in motor neurons compared to iPSCs, whereas 1B-transcripts remained relatively stable between iPSCs and motor neurons (Fig. 4D).

### Knock-out validation of commercial C9orf72 antibodies for Western blot

**Figure 5:**
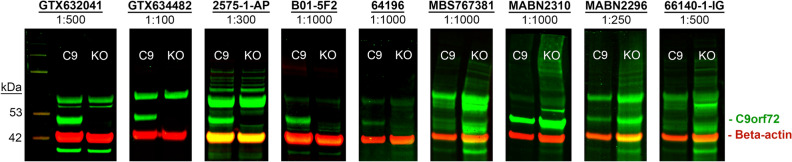
Knock-out validation of commercial C9orf72 antibodies indicated for Western blot. Immunofluorescent Western blot images showing nine commercial C9orf72 antibodies tested at concentrations recommended by the manufacturer. A band of ~ 52 kDa corresponding to C9orf72 (in green) was present in 2-week-old iPSC-derived motor neurons from a C9orf72 ALS/FTD patient, but absent in the KO line only for antibodies GTX632041, GTX634482, 2575-1-AP and B01-5F2. C9 = Pooled technical replicates (wells) of 2-week-old C9 ALS/FTD patient iPSC-derived motor neurons. KO = Pooled technical replicates (wells) of 2-week-old homozygous KO iPSC-derived motor neurons. Membranes were co-incubated with beta-actin loading control (42 kDa, in red).

We tested the specificity of commercially available antibodies against C9orf72 in 2-week-old patient iPSC-derived motor neurons using homozygous KO iPSC-derived motor neurons as a negative control. We generated a homozygous KO of the *C9orf72* gene in a patient iPSC line harboring approximately 200 repeats, using bi-allelic excisions starting upstream of exon 1A and ending in exon 3. Of 9 commercially available Western blot-indicated primary antibodies (Table S2), including 5 antibodies not previously tested in systematic KO-validation screens^14,30^, we found 4 antibodies to be specific for C9orf72 in motor neuron lysates at supplier-indicated concentrations: GTX632041, GTX634482, 2575-1-AP and B01-5F2 (Fig. 5). The other 5 antibodies showed either no signal or equivalent signal in KO motor neurons.

### Lack of specificity of commercial C9orf72 antibodies for immunocytochemistry in iPSC-derived motor neurons

To test the specificity of antibodies for C9orf72 when applied to immunocytochemistry, we used the previously described 200-repeat C9-patient line and isogenic homozygous *C9orf72* KO line and generated a further homozygous *C9orf72* KO line on our human WT line background, using bi-allelic excisions starting upstream of exon 1A and ending in exon 2. We were interested in understanding the spatial localization of the C9orf72 protein in iPSC-derived motor neurons, but unfortunately, after testing 9 commercially available antibodies for immunocytochemistry, we found none that were specific to C9orf72: they either showed no signal in any line or showed signal in our two KO lines (Fig. S2, Table S2).

### Identification of antibodies specific to dipeptide repeat proteins in patient-derived iPSC-motor neurons, patient postmortem brain, *C9orf72* mutant BAC transgenic mouse brain

**Figure 6:**
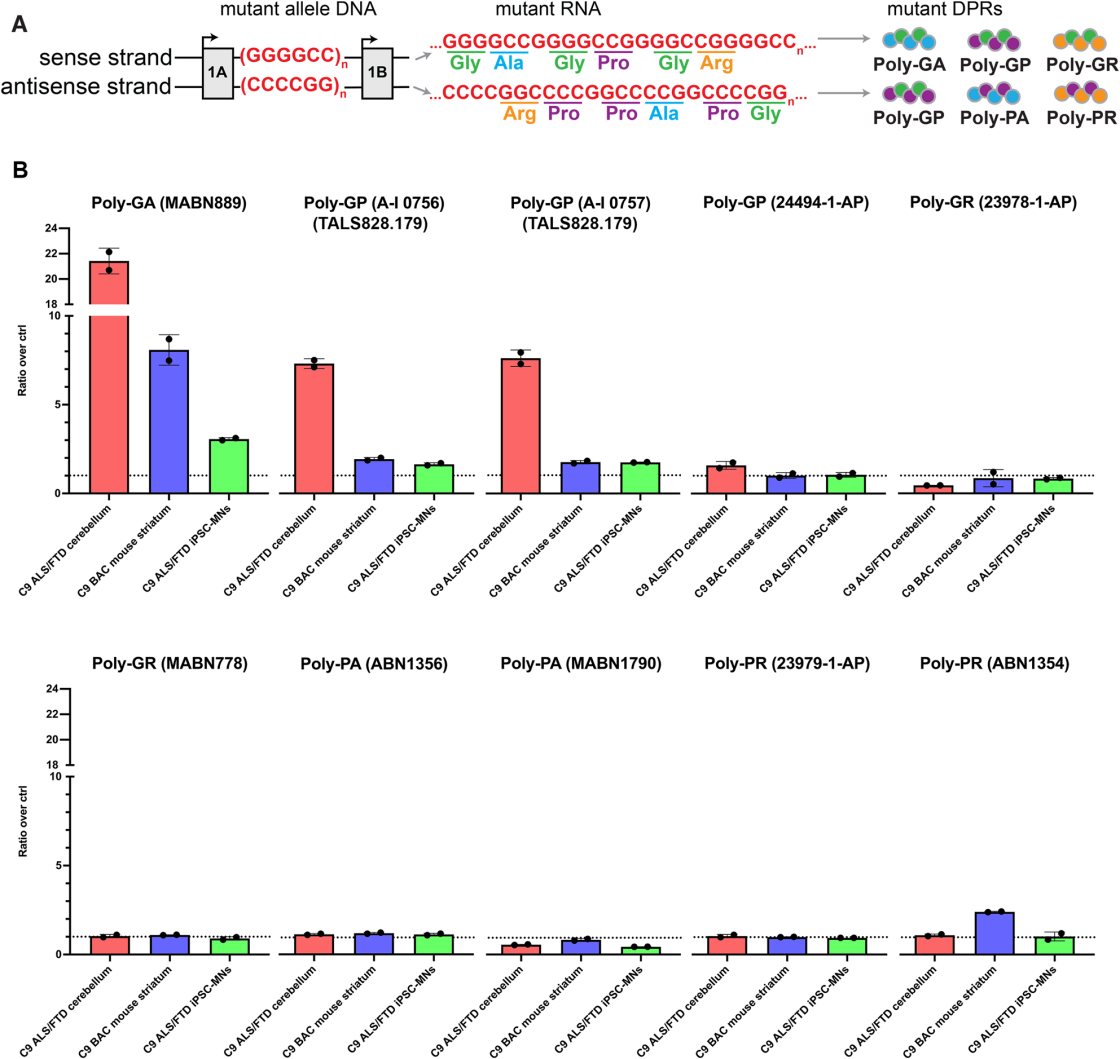
Specificity of commercial dipeptide repeat protein antibodies on MSD immunoassay. (**A**) Schematic of sense and antisense RNAs carrying the repeat expansion and of their translation through non-canonical repeat-associated non-AUG (RAN) translation. RAN translation is expected to produce 5 different dipeptide repeat proteins (DPRs): poly-GA and polyGR from the sense strand, poly-PA and poly-PR from the antisense strand and poly-GP from both the sense and antisense strands. (**B**) Ten DPR antibodies were tested on MSD immunoassay in 3 sample types: Human C9-ALS/FTD post-mortem cerebellum versus non-mutant neurologically unaffected post-mortem cerebellum, *C9orf72* mutant transgenic mouse striatum versus WT mouse striatum, and 2-week-old C9-ALS/FTD patient iPSC-derived motor neurons (MNs) versus isogenic 2-week-old *C9orf72* KO iPSC-MNs. Further information on tissues used are provided in Table S3. Electrochemiluminescence signals were normalized to background signals from negative controls of the same sample type, producing signal ratios shown on the y-axis. Raw signals are reported in Table S4. Poly-GA antibody MABN889 and lots A-I 0756 and A-I 0757 of poly-GP antibody TALS828.179 selectively recognized poly-GA and poly-GP respectively in all sample types assessed. Dotted line = ratio of 1, i.e. no change compared to control. N = 2 technical replicates. Error bars = SD.

*C9orf72* is transcribed off of both the sense and antisense strands in normal and diseased cells (Fig. 6A)^7,31–33^. In diseased cells, some transcripts retain their intronic repeat expansion, which can undergo non-canonical RAN translation, leading to the production of two amino acid strings called dipeptide repeat (DPR) proteins^4–13^. DPRs result from translation in any open reading frame from the sense or antisense strand, including poly-GA and poly-GR from the sense strand; poly-PA and poly-PR from the antisense strand; and poly-GP from both the sense and antisense strands (Fig. 6A).

Many of the antibodies used to quantify DPRs have been raised against or validated through positive selection to recombinant protein^6,9,10,34^. Negative validation is important because the two amino acid combinations present in the DPRs are also common in other peptides. We designed an assay using Meso Scale Discovery’s (MSD) electrochemiluminescence-based sandwich immunoassay to evaluate the specificity of commercially available antibodies or antibodies available through the TargetALS consortium. We evaluated 10 antibodies, across various combinations of 8 conditions (capture antibody identity and concentration, detection antibody identity and concentration, lysate concentration, plate type, blocking buffer and diluent solution (TBS vs PBS)) (Fig. S3). We assessed these variables to increase the likelihood of DPR detection, with a total of 60 tested conditions. We first compared signals in 2-week-old motor neurons induced from our patient iPSC line harboring ~ 200 repeats to those of motor neurons induced from the isogenic *C9orf72* KO line. A signal ratio of 1 between the unedited and KO lines indicates equal (and therefore nonspecific) signal, due to either equally low or high signal in both lines. A ratio > 1 demonstrates signal specific for the DPR. We found 2 antibody combinations (against poly-GA and poly-GP) that produced a greater than twofold signal in the unedited patient versus KO line (Fig. S3, green highlights), and therefore could detect the presence of DPRs above the background signal defined by our KO line.

Because low-abundance arginine-rich DPRs may not be detectable in all patient-derived iPSC lines, we next validated our findings in tissue lysates from human C9-ALS postmortem cerebellum and *C9orf72* mutant BAC transgenic mouse striatum (Table S3), using optimized parameters from our initial optimization assays. Signal ratios were calculated by dividing signals from repeat-expansion containing tissues with signals from non-diseased tissue-matched negative controls. Poly-GA antibody MABN889 and poly-GP antibody TALS828.179 (lots A-I 0756 and A-I 0757) showed specificity with a ratio greater than 1.5 across all sample types (Fig. 6B, Table S4). Under our assay parameters, no other antibody showed specificity (i.e., had signal in C9-lysate greater than signal in KO/WT lysate) across samples*.* Interestingly, poly-PR antibody ABN1354 displayed a signal ratio of 2.4 in transgenic compared to WT control mouse brain, but not in human postmortem tissue or iPSC-derived motor neurons. In summary, we confirmed the specificity of 2 commercially available DPR antibodies against poly-GA and poly-GP across different C9-sample types.

## Discussion

*C9orf72* is a particularly challenging gene to work with because the repetitive DNA of the mutation and the mutant allele’s products are hard to measure. Here we share our efforts to create human-specific validated assays to measure *C9orf72* DNA, mRNA and dipeptide repeats using patient-derived iPSC lines.

Measuring the length of the *C9orf72* repeat expansion accurately is important for a number of reasons. Current methods used to clinically diagnose *C9orf72* repeat expansion tell us that the region is expanded, but do not size long repeats (i.e. repeat-primed PCR (GS/RP-PCR), which lacks accuracy) or require high DNA input and fail to detect rare repeats or repeats of mixed sizes (i.e. Southern blot, which lacks sensitivity). We demonstrate that long range, single-molecule sequencing can accurately size the repeat expansion across a range of repeat lengths up to the thousands, with less DNA input than required for Southern blot and increased accuracy compared to GS/RP-PCR (Fig. 1). Additionally, single molecule sequencing could foster greater reproducibility of repeat expansion sizing across labs or genetic backgrounds, which has been a challenge in the field^35^. In particular, because single-molecule sequencing does not require primer or probe binding (as PCR and Southern blot do, respectively), it can still perform when genetic variation (such as indels) is present around the repeat expansion. With this tool, we can now address important biological questions about *C9orf72* pathobiology, including whether mosaicism in repeat lengths exists across cell types and whether or not the repeat expansion is stable over time in one individual or cell population.

Our approach builds upon the success of long-range sequencing to size the repeat expansion from plasmid^18^ and human tissue^19,20^, and demonstrates its usefulness to additional applications, in particular in the context of CRISPR gene editing. We show that using SNPs, we can phase the repeat expansion to allele-specific sequences, and use single-molecule long-range sequencing to assess our edits on each allele separately (Fig. 3). Furthermore, we show that CRISPR editing can result in mixed clones with multiple editing outcomes. We suspect mixed clones, including some that retained an intact repeat expansion, have occurred in prior CRISPR editing experiments, and have confounded the analysis of these edits. With single molecule sequencing, we show that we can determine the clonality of a cell line. Moreover, we can quantify editing outcomes that differ by only a few nucleotides, showing that this method can be used to characterize small insertions or deletions (indels) induced at cut sites by CRISPR editing.

Distinguishing the transcripts emanating from *C9orf72*’s multiple start sites is important to understand the biology of the locus. For instance, the Genotype-Tissue Expression Project (GTEx)^36^ shows that *C9orf72* is expressed throughout the body, yet the mutant repeat expansion only seems deleterious in the central nervous system. Using ddPCR probes specific to exon 1A or 1B and cell lines with bi-allelic excisions of these exons, we uncovered interesting aspects of gene regulation at the *C9orf72* locus. First, excision of exon 1A decreased the expression of 1B-containing transcripts in iPSC-derived motor neurons. Second, 1A-containing transcripts are nearly undetectable in iPSCs, but are upregulated 10–15× in iPSC-derived motor neurons. In contrast, 1B-containing RNAs constitute the majority of transcripts and their expression levels remain constant between iPSCs and induced motor neurons. Since sense expression of the repeat expansion is driven by exon 1A-transcription (and not exon 1B), the upregulation of exon 1A transcripts in motor neurons may explain why the repeat expansion manifests as a neurologic disease. In the future, our ddPCR probes could be used on human samples to determine whether 1A transcripts are specific to the subset of neurons vulnerable to ALS/FTD neurodegeneration.

How exactly expanded repeats cause toxicity is still debated, but it is clear that the *C9orf72* repeat expansion encodes DPRs that can be produced by non-conventional translation off of both strands, and that these DPRs are likely to be toxic to cells. It is therefore important to distinguish and quantify these DPRs, to locate them in the cell and to determine whether genetic manipulations can eliminate them. It is also important to detect and localize C9orf72 protein, and to determine how its production is affected by the repeat and genetic manipulations aimed at neutralizing it, or by genetic background. It is disappointing that we did not find any current-generation C9orf72 antibodies that were specific for immunocytochemistry in iPSC-derived motor neurons (Fig. S2), extending prior work that also noted a lack of specificity of many C9orf72 antibodies^14,30^. Nevertheless, we succeeded in identifying 4 commercial antibodies specific for C9orf72 protein via Western blot, additionally validating a previous report^14^ (Fig. 5). We limited our survey of Western blot-indicated commercial C9orf72 antibodies to those with evidence from publication or claim from the manufacturer of knock-out validation. Given the observed differences in antibody performance at supplier-indicated concentrations, we recommend knock-out validation of newly developed antibodies. Contrary to previous reports, we did not observe that a high amount of protein was necessary to detect C9orf72 in our iPSC-derived motor neuron lysates, as we loaded 17 µg protein versus 50 µg as previously reported^14,30^. Importantly, we also identified two commercial antibodies against poly-GA and poly-GP (Fig. 6B) appropriate for DPR quantification by MSD immunoassay. We caution the field that the C9orf72 and DPR antibodies that show high signal in KO lines may have led to spurious results and recommend that KO lines be used for validation of all antibodies in the future.

There are important limitations to the methods we have described. The first is that the number of sequencing reads decreases as the repeat expansion increases. Even with this limitation we were able to detect mixed clones with mutations up to 1500 repeats (~ 9 kb). We expect that this limitation will keep receding as read depth continues to increase and the cost of sequencing decreases. Secondly, we were unable to detect the short version of *C9orf72* 1A transcripts (1A-short, or variant 1 transcripts) in iPSC-derived motor neurons. As a result, we cannot vouch for the specificity of our probe for 1A-long versus 1A-short RNAs. The 1A-short transcript appears to be expressed at low levels in brain tissue and may be altered by the mutation. Because the 1A-long (variant 3) and 1A-short (variant 1) overlap in exon 1A, validating probe specificity will require more selective genetic knockouts than those we performed here. Alternatively, more mature or aged cell culture systems may allow us to detect 1A-short transcripts in vitro*.* Thirdly, neither ddPCR nor any current sequencing method can directly measure RNA containing the repeat expansion, because amplification fails across the repeat expansion. Perhaps no-amplification RNA sequencing methods, similar for the methods we’ve used on DNA, would resolve the issue. Finally, while we have validated antibodies for poly-GA and poly-GP quantification, other DPRs are expected from endogenous RAN translation of the sense and antisense repeat-containing transcripts. At the moment, we cannot fully rule out that our failure to detect additional DPRs meant that current antibodies are not specific enough, or that our assay conditions (e.g. protein and antibody concentrations or sample preparation) were not optimal for detection of poly-GR, poly-PR and poly-PA, which are the least abundant of the five DPR species under endogenous RAN translation.

In summary, we offer validated methods for the quantification of C9orf72 at the DNA, RNA and protein levels. We recommend that RNA and protein quantification assays be validated in C9orf72 KO lines. In addition, we recommend single-molecule sequencing as the gold standard for verifying unedited and edited cell lines, since it can accurately size the repeat expansion, report on clonality, phase surrounding polymorphisms and determine editing outcomes (conflict of interest declaration: two of the authors work for PacBio with one holding company stocks; the academic authors who independently validated the assays described here made this recommendation). Adoption of these or similar measures will ensure transparency and reproducibility in the field.

## Methods

### Cell line maintenance

We used iPSC generated by others^21,23,24^ from patients harboring the *C9orf72* mutation and a control cell line without mutation^37^ (WT-control). We maintained iPSCs in mTesR plus plated on Matrigel (Corning 356231), passaging at 60–80% confluency. All cell lines had a normal karyotype and negative quarterly mycloplasma testing.

### PacBio single molecule sequencing to size the repeat expansion and detect repeat expansion excision

Because polymerase amplification fails to accurately size the entirety of the *C9orf72* GC-rich repetitive region, we used single molecule sequencing^18,38^ of a genomic region containing the repeat region. We collected high-molecular-weight DNA using Genomic Tip (Qiagen 10243) and confirmed absence of smearing by running the DNA on a 1.5% agarose gel. The Gladstone Genomics Core performed library preparation according to the “No Amp Targeted Sequencing” published protocol^18,39^ using 3–5 µg of DNA per sample as measured by Qubit. Briefly, we blocked the free ends of purified genomic DNA and then excised the gene region of interest using spCas9, a gRNA targeting 5′ to the repeat expansion (GGAAGAAAGAATTGCAATTA) and a gRNA targeting 3′ to the repeat expansion (TTGGTATTTAGAAAGGTGGT). Excising the genomic region harboring the repeat expansion yields a 3639 bp fragment from the WT allele and a fragment of variable size from the mutant allele depending on the size of the GGGGCC repeat. We then ligated adapters and barcodes to blunt free ends and sequenced 3–5 barcoded lines per SMRT Cell on either a Sequel I or Sequel II sequencer. We used a 3-pass filter such that each molecule of DNA had to be sequenced at least 3 times to be included in analysis. We compared repeat counts from sequencing to Southern blot, performed by Celplor using 20 µg of input DNA and the previously published protocol^16^, and GS/RP-PCR, performed by Asuragen using 20–80 µg of input DNA and the previously published protocol^15^.

### iPSC line CRISPR engineering

Before generating the 1Ax, 1Bx and KO isogenic series in our WT iPSC^37^ line, we first knocked-in the inducible motor neuron transcription factor transgene cassette^27,28^ in the CLYBL safe-harbor locus using spCas9 and ATGTTGGAAGGATGAGGAAA gRNA. This transgene includes human NGN2, ISL1, LHX3 (hNIL) under the TET operator and is inducible by doxycycline. The transgene also drives mCherry (for positive selection) and neomycin antibiotic resistance (for negative selection). Red-fluorescing cells were sorted via FACS to isolate single, live cells. Each resulting clonal cell line was analyzed for incorporation of the transgene in the CLYBL locus by PCR (left homology arm junction primers CAGACAAGTCAGTAGGGCCA and AGAAGACTTCCTCTGCCCTC) with preservation of one of the alleles (CLYBL wild-type primers TGACTAAACACTGTGCCCCA and AGGCAGGATGAATTGGTGGA). We used Copy Number Variation (CNV) ddPCR to pick a clone with a single transgene insertion of the hNIL plasmid (Nemomycin primers CATGGCTGATGCAATGCG and TCGCTTGGTGGTCGAATG, probe FAM; Primers UBE2D2—Bio-Rad 10031255, probe HEX) to mitigate the risk of integration of the transgene at genomic loci other than CLYBL.

For iPSC CRISPR engineering we used HiFi spCas9 protein (Macolabs, UC Berkeley) and two gRNAs to create an excision, using our published protocol^40^. The gRNA pairs are listed in Table S5. gRNAs were designed to have no exact off-target matches and the lowest predicted off-targets using CRISPOR (Homo sapiens—USCS Dec. 2013 (GRCh38/hg38))^41^. gRNAs were ordered from IDT or Synthego. Cas9-gRNA RNP (spCas9 (40 µM), sgRNA (100 µM)) was delivered by nucleofection (Lonza AAF-1002B, Lonza AAF-1002X, Pulse Code = DS138) to 350,000 iPSCs suspended in 20 µl of P3 Buffer. The cells were recovered with mTesR plus supplemented with ROCK1 inhibitor (Selleckchem S1049) at 10 µM and Clone R (Stemcell 05888). Approximately 50% of iPSCs died within the first 24 h of electroporation, as expected. Following a 48–72-h recovery, we collected the pool of edited cells and either hand-picked 48 clones or sorted single live cells via FACS to a single well on a 96-well plate. Single-cell sorting was performed using a BD FACSAria Fusion (Beckton Dickinson) by the Gladstone Flow Cytometry Core. The QC alignment of each laser was verified with Cytometer Setup and Tracking Beads (Becton Dickinson) before sample acquisition. A forward scatter threshold of 15,000 was set to eliminate debris from list mode data, and a fixed number of events was collected. In some experiments mCherry fluorescence (excitation 561 nm, emission 610 nm) was also used to define sorting parameters. Drop delay determination and 96-well plate setup was done using Accudrop beads (Becton Dickinson). Gating on forward scatter area versus height and on side scatter area versus height was used to make the single cell determination. The specifications of the sort layout included single cell precision, 96-well collection device and target event of 1. After cultures reached 60–70% confluency, each well was split into two wells of a new 48- or 96-well plate, one for sequencing and the other to continue the cell line. We screened clones based on the presence of an excision band using PCR (primers and expected band size from Table S5. We also performed PCR across each the 5′ and 3′ cut site (Table S5), with one primer site located inside the excision region, to ensure absence of a band (for homozygous edits). For all lines except the patient repeat expansion excision line, we then Sanger sequenced the excision band (MCLAB, Quintara). If the sequence was ambiguous (i.e., had overlapping nucleotide reads at the same mapped nucleotide position) we subcloned the line to achieve clone purity and clean sequencing. All lines were karyotyped (WiCell or Cell Line Genetics) after editing.

### iPSC differentiation into motor neurons

We used the hNIL transgene cassette TET-on system in the CLYBL safe-harbor locus of a C9-patient line and WT-control line. Introduction of doxycycline for 3 days induced the expression of 3 human transcription factors: NGN2, ISL1, LHX3. We followed the previously published protocol^27,28^ with notable exceptions, including higher concentrations of the growth factors BDNF, GDNF and NT-3 (each at 20 ng/ml). Our detailed protocol is published^42^.

### RNA quantification by ddPCR

2-week-old induced neurons were lysed with papain (Worthington LK003178) and RNA was isolated using Quick-RNA Microprep Kit (Zymo R1051). cDNA was synthesized using iScript™ Reverse Transcription Supermix (Biorad 1708841) from 500 ng of RNA. ddPCR was run with 3 technical replicates of each of 3 biologic replicates (independent wells of differentiated neurons) on the QX100 Droplet Reader (Bio-Rad 186-3002). Each ddPCR reaction consisted of 12.5 µL of 2× SuperMix for Probes (no dUTP) (Bio-Rad 186-3024), primer/probe (see Table S1), 5 ng of cDNA, and nuclease-free water up to 25 µL. Droplets were generated with QX 100 Droplet Generator (Bio-Rad 186-3001) and 20 µL of the reaction mixture with 70 µL of oil. The ddPCR reactions were run in a Deep Well C1000 Thermal Cycler (Bio-Rad 1851197) with the following cycling protocol: (1) 95 °C for 10 min; (2) 94 °C for 30 s; (3) 58 °C for 1 min; (4) steps 2; and 3 repeat 39 times; (5) 98 °C for 10 min; (6) hold at 4 °C. We thresholded positive samples as those with > 10 positive droplets to avoid error due to noise. We quantified positive droplets for each target and normalized the amount to our loading control (*UBE2D2*) (Bio-Rad QuantaSoft™ Analysis Pro Software). We chose this housekeeping gene because its expression level remained stable across iPSCs and differentiated neurons^43^.

### C9orf72 immunoassays

For immunocytochemistry, 2-week-old iPSC-derived motor neurons were fixed by adding 4% PFA directly to culture media for 30 min followed by 3 PBS washes of 10 min each. Cells were permeabilized by 1X DPBS 0.1% Triton-X in 3 washes of 10 min each at room temperature and blocked with 1X DPBS 0.1% Triton-X + 5% BSA for 1 h at room temperature. Primary antibodies (concentrations reported in Table S2) were incubated overnight at 4 °C. Secondary antibodies (Table S2) were incubated at room temperature for 1 h. DAPI (D1306, ThermoFisher Scientific) was added to the penultimate of five, 5 min PBS washes. Our detailed immunocytochemistry protocol is published^44^.

For Western blot, 2-week-old iPSC-derived motor neurons were lysed in RIPA buffer (89900, Pierce) with 3X Halt Protease and Phosphatase Inhibitor Cocktail (78441, Thermo Scientific) and prepared for SDS-PAGE electrophoresis by boiling for 5 min at 95 °C in NuPAGE LDS Sample Buffer (NP0007, Invitrogen) and NuPAGE Sample Reducing Agent (NP0009, Invitrogen). 17 µg of protein was loaded per well. Next, the separated proteins were transferred to nitrocellulose membranes via iBlot 2 rapid transfer device (Invitrogen), followed by blocking either in 3% skim milk powder in PBS + 0.1% Tween-20 (PBS-T), or Intercept PBS Blocking Buffer (927-70001, Licor) for 1 h at room temperature. Membranes were incubated with C9orf72 antibodies diluted in blocking buffer overnight at 4 °C. C9orf72 primary antibodies were diluted to concentrations indicated for Western blot by the supplier (Table S2). Beta-actin primary antibodies (Table S2) were used 1:1000 and incubated for 1.5 h at room temperature. Following primary antibody incubation, membranes were washed 5 times for 5 min each in PBS-T, followed by secondary antibody (detailed in Table S2) incubation at room temperature for 1 h. Next, membranes were washed 5 times for 5 min each in PBS-T and imaged on an Odyssey M Imaging System (Licor). Uncropped Western blots are shown in Fig. S4.

### Dipeptide repeat protein quantification by Meso Scale Discovery (MSD) immunoassay

We followed the manufacturer’s protocol for the Small Spot Streptavidin Plate (L45SA, MSD). Antibodies, concentrations and reagents used are detailed in Figs. 6B and S3 and Table S2. The plate was coated with biotinylated capture antibody overnight at 4 °C without agitation, following a 15 min agitation at room temperature at 600 rpm. The plate was blocked with 3% MSD Blocker A (R93BA, MSD) or skim milk powder in 1X DPBS for 1 h at 600–750 rpm, then incubated for 1–2 h with protein lysates at 600–750 rpm at room temperature or at 4 °C. Next, SULFO-TAG labeled detection antibody was added for 1 h at room temperature at 600–750 rpm. Washes were performed between steps thrice with 1X DPBS + 0.05% Tween-20. MSD Read Buffer A (R92TG, MSD) was added to the plate before immediate reading on an MSD Model 1250 Sector Imager 2400 plate reader. Signal ratios were calculated by dividing electrochemiluminescence intensities of positive control samples with background intensities defined by KO or WT negative control samples of the same sample type. Data were presented as fold changes (ratios) above baseline (background) level. Our detailed MSD immunoassay protocol is published^45^.

### Supplementary Information

Supplementary Information.

## Data Availability

Single molecule sequencing data are available at NIH Bioproject, https://www.ncbi.nlm.nih.gov/bioproject/PRJNA1006439.
