# Supplementary material for: Validated assays for the quantification of C9orf72 human pathology
Source: Sci Rep. 2024 Jan 8;14:828. doi: 10.1038/s41598-023-50667-3 (PMC10774390; doi:10.1038/s41598-023-50667-3)
Supplement: Supplementary file 1 — Supplementary Information. [file 41598_2023_50667_MOESM1_ESM.pdf]

Supplementary Figures and Tables

Validated assays for the quantification of C9orf72 human pathology

Salomonsson SE*[1,2], Maltos AM*[3], Gill K[1,2,3], Aladesuyi Arogundade O[1,2], Brown KA[1,2], Sachdev A[3], Sckaff M[1,2,3], Lam KJK[1,2], Fisher IJ[1,2], Chouhan RS[1,2], Van Laar VS[4,5], Marley CB[1], McLaughlin I[6], Bankiewicz KS[4,5], Tsai Y-C[6], Conklin BR[3,7], Clelland CD[#1,2].

[1]Weill Institute for Neurosciences, University of California San Francisco, San Francisco, California, USA
[2]Memory & Aging Center, Department of Neurology, University of California San Francisco, San Francisco, California, USA
[3]Gladstone Institutes, San Francisco, California, USA
[4]Department of Neurological Surgery, The Ohio State University, Columbus, Ohio, USA
[5]The Gene Therapy Institute, The Ohio State University, Columbus, Ohio, USA
[6]Pacific Biosciences, Menlo Park, California, USA
[7]Departments of Medicine, Ophthalmology, and Pharmacology, University of California San Francisco, San Francisco, California, USA.
*These authors contributed equally
#Correspondance: Claire D. Clelland: claire.clelland@ucsf.edu

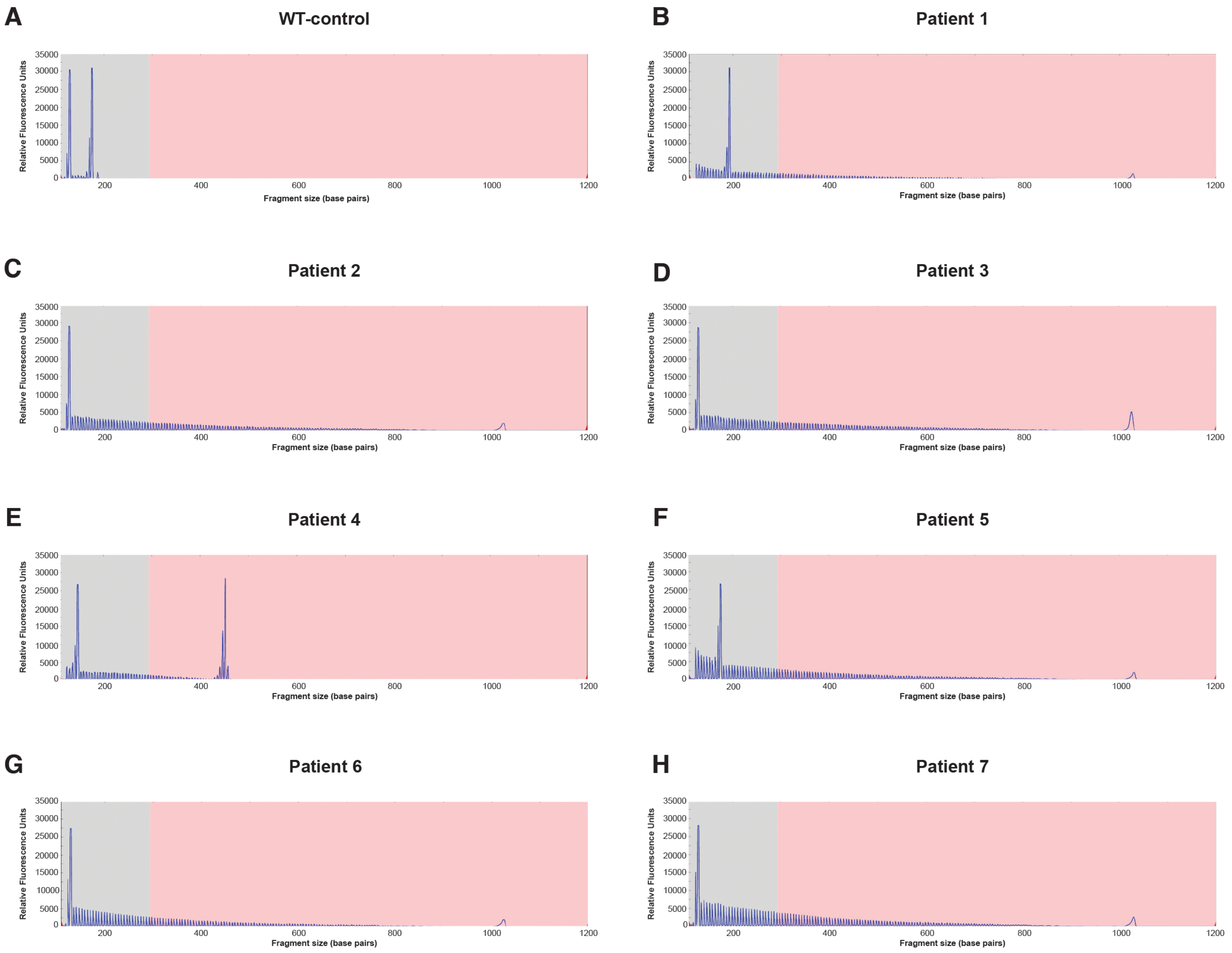
A
WT-control
B
Patient 1
C
Patient 2
D
Patient 3
E
Patient 4
F
Patient 5
G
Patient 6
H
Patient 7
Relative Fluorescence Units
35000
30000
25000
20000
15000
10000
5000
0
200
400
600
800
1000
1200
Fragment size (base pairs)

Supplemental Figure 1. Electropherograms for each patient line produced by GS/RP-PCR and capillary electrophoresis. (A-H) Gene specific, repeat primed PCR detects *C9orf72* repeats up to 145. Each patient has one non-expanded allele (left blue peak in the grey region). Expanded repeats less than 145 (*e.g.,* Patient 4, right blue peak) are accurately sized, but there is no quantification of repeats greater than 145 (lack of equivalent second blue peak for every other patient). Instead, the expanded allele is detected but not sized by the presence of the small peak around position 1000 (~140 repeats, pink region).

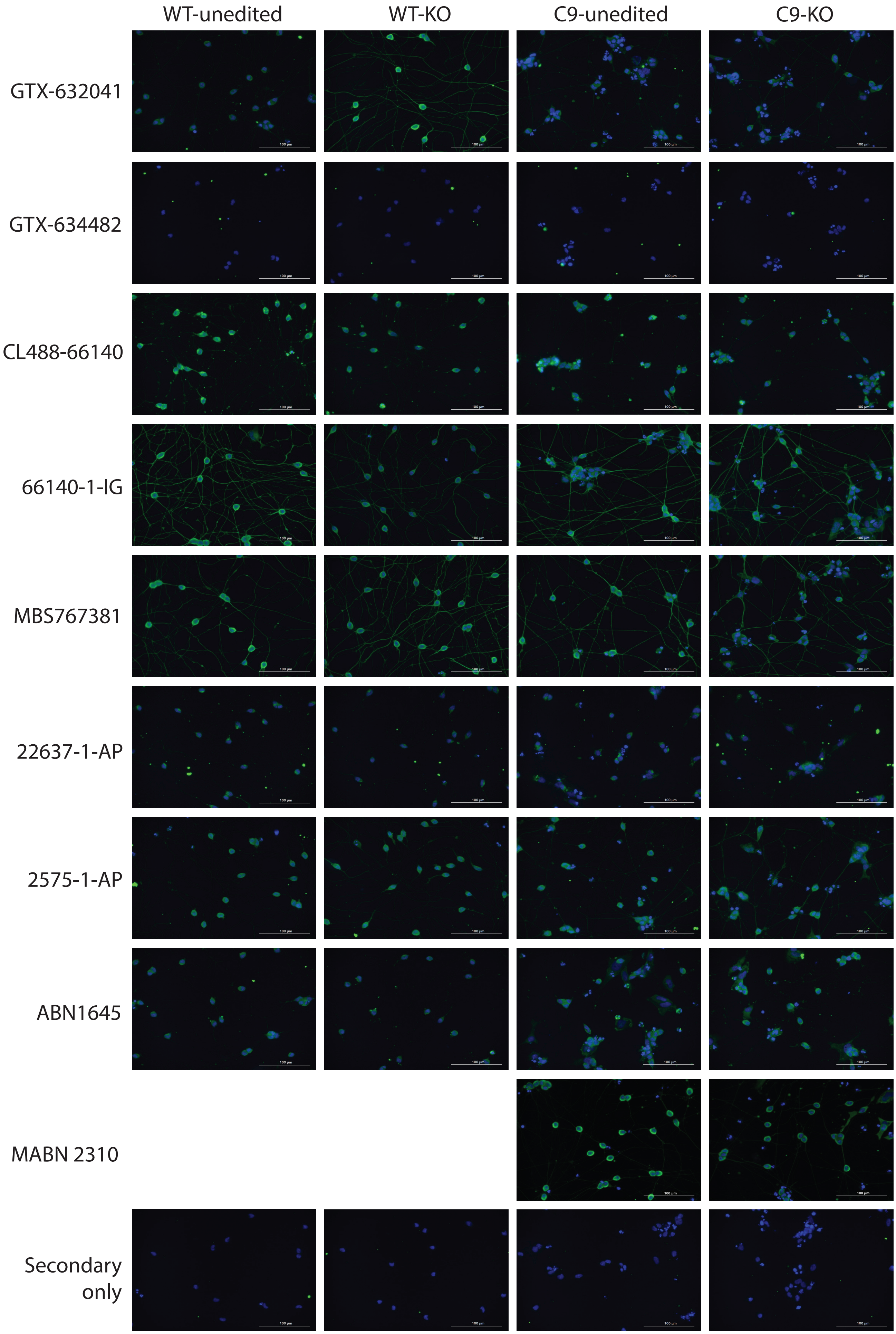
WT-unedited
WT-KO
C9-unedited
C9-KO
GTX-632041
GTX-634482
CL488-66140
66140-1-IG
MBS767381
22637-1-AP
2575-1-AP
ABN1645
MABN 2310
Secondary only

Supplemental Figure 2. Nine commercial C9orf72 antibodies were not specific for C9orf72 in iPSC-derived motor neurons by immunocytochemistry. Commercially available C9orf72 antibodies were not specific for C9orf72, as shown by comparing staining patterns in knock-out lines (WT-KO and C9-KO) to unedited cells (WT-unedited and C9-unedited). Blue = DAPI. Green = staining with antibodies listed in Table 2. Scale bar = 100 μm.

A

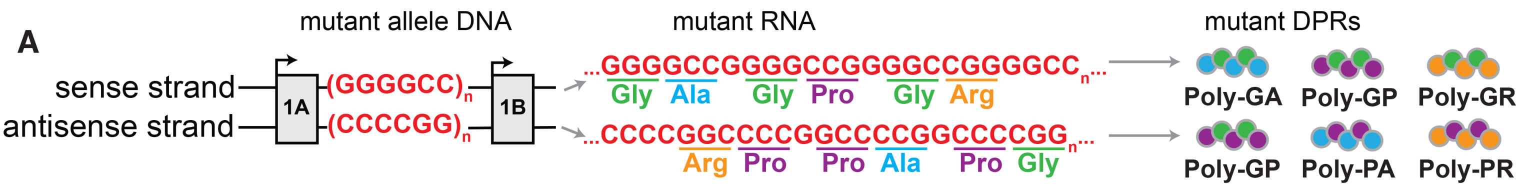

B

DPR	Capture	Detect	Capture Concentration (µg/mL)	Detect Concentration (µg/mL)	Lysate Concentration (µg)	Plate	Blocker	Solution	C9/KO Ratio
Poly - GA	MABN889	MABN889	1	1	18	Small Spot Streptavidin	Blocker A	PBS	17.4
				2	18	Small Spot Streptavidin	Blocker A	PBS	20.4
			4	1	18	Small Spot Streptavidin	Blocker A	PBS	16.3
				2	18	Small Spot Streptavidin	Blocker A	PBS	14.44
Poly - GP	24494-1-AP	24494-1-AP	1	1	9.37	Standard	Blocker A	PBS	1.3
					18.75	Standard	Blocker A	PBS	1.6
					37.5	Standard	Blocker A	PBS	2
				2	9.37	Standard	Blocker A	PBS	1.6
					18.5	Small Spot Streptavidin	Blocker A	PBS	1.27
							Milk	PBS	1.26
						Standard	Milk	PBS	1.33
					18.75	Standard	Blocker A	PBS	1.7
					25	Small Spot Streptavidin	Blocker A	PBS	0.99
					37.5	Standard	Blocker A	PBS	1.9
					20 (Denatured)	Small Spot Streptavidin	Blocker A	PBS	1.34
								TBS	0.85
			4	1	9.37	Standard	Blocker A	PBS	1.4
					18.75	Standard	Blocker A	PBS	1.6
					37.5	Standard	Blocker A	PBS	1.5
				2	9.37	Standard	Blocker A	PBS	1.2
					18.75	Standard	Blocker A	PBS	1.3
					37.5	Standard	Blocker A	PBS	1.5
	A-I 0756	A-I 0756	2	2	18.5	Small Spot Streptavidin	Blocker A	TBS	2.4
		A-I 0757	2	4	18.5	Small Spot Streptavidin	Blocker A	TBS	2.63
	A-I 0757	A-I 0756	2	2	18.5	Small Spot Streptavidin	Blocker A	TBS	2.01
		A-I 0757	2	4	18.5	Small Spot Streptavidin	Blocker A	TBS	2.3
Poly - GR	23978-1-AP	23978-1-AP	1	1	18.5	Small Spot Streptavidin	Blocker A	PBS	0.94
				2	18.5	Small Spot Streptavidin	Blocker A	PBS	0.83
			4	1	18.5	Small Spot Streptavidin	Blocker A	PBS	0.91
				2	18.5	Small Spot Streptavidin	Blocker A	PBS	1.01
		MABN778	2.33	2	20	Small Spot Streptavidin	Blocker A	PBS	0.92
	MABN778	23978-1-AP	1	2	20 (Denatured)	Small Spot Streptavidin	Blocker A	PBS	0.77
			2.33	2	20	Small Spot Streptavidin	Blocker A	PBS	0.99
		MABN778	1	1	18	Small Spot Streptavidin	Blocker A	PBS	0.84
				2	18	Small Spot Streptavidin	Blocker A	PBS	0.9
					20 (Denatured)	Small Spot Streptavidin	Blocker A	PBS	0.77
			4	1	18	Small Spot Streptavidin	Blocker A	PBS	0.95
				2	18	Small Spot Streptavidin	Blocker A	PBS	1
Poly - PA	ABN1356	ABN1356	1	1	18.5	Small Spot Streptavidin	Blocker A	PBS	0.86
				2	18.5	Small Spot Streptavidin	Blocker A	PBS	0.88
					45	Small Spot Streptavidin	Blocker A	PBS	0.9
			4	1	18.5	Small Spot Streptavidin	Blocker A	PBS	0.86
				2	18.5	Small Spot Streptavidin	Blocker A	PBS	0.98
	MABN1790	MABN1790	1	1	18.5	Small Spot Streptavidin	Blocker A	PBS	1.11
				2	18.5	Small Spot Streptavidin	Blocker A	PBS	0.99
					45	Small Spot Streptavidin	Blocker A	PBS	0.93
			4	1	18.5	Small Spot Streptavidin	Blocker A	PBS	1.12
				2	18.5	Small Spot Streptavidin	Blocker A	PBS	1.03
Poly - PR	23979-1-AP	23979-1-AP	1	1	18.5	Small Spot Streptavidin	Blocker A	PBS	0.97
				2	18.5	Small Spot Streptavidin	Blocker A	PBS	0.93
					45	Small Spot Streptavidin	Blocker A	PBS	0.84
			4	1	18.5	Small Spot Streptavidin	Blocker A	PBS	0.91
				2	18.5	Small Spot Streptavidin	Blocker A	PBS	0.93
		ABN1354	2.33	2	20	Small Spot Streptavidin	Blocker A	PBS	1.07
	ABN1354	23979-1-AP	2.33	2	20	Small Spot Streptavidin	Blocker A	PBS	0.97
		ABN1354	1	1	18	Small Spot Streptavidin	Blocker A	PBS	0.88
				2	18	Small Spot Streptavidin	Blocker A	PBS	0.96
					45	Small Spot Streptavidin	Blocker A	PBS	0.95
			4	1	18	Small Spot Streptavidin	Blocker A	PBS	1.03
				2	18	Small Spot Streptavidin	Blocker A	PBS	0.97

Supplemental Figure 3. **Two of 10 antibodies tested were specific for dipeptide repeat proteins in *C9orf72* mutant iPSC-derived motor neurons compared to *C9orf72* KO control.** **(A)** Schematic of sense and antisense RNAs carrying the repeat expansion and their translation through non-canonical repeat-associated non-AUG (RAN) translation. RAN translation is expected to produce 5 different dipeptide repeat proteins (DPRs): poly-GA and poly-GR from the sense strand, poly-PA and poly-PR from the antisense strand and poly-GP from both the sense and antisense strands. **(B)** We tested 10 DPR antibodies under various conditions using MSD immunoassay. Concentrations of capture and detection antibodies and lysate concentrations from 2-week-old iPSC-motor neurons are noted. We compared a C9-ALS/FTD patient line harboring ~200 repeats to an isogenic *C9orf72* KO line. Most antibodies generated signals similar between KO and C9-ALS/FTD patient lines (= signal ratio near 1).

GTX632041
1:500

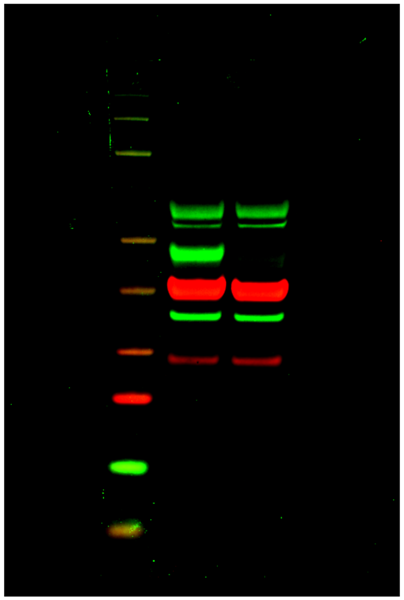

GTX634482
1:100

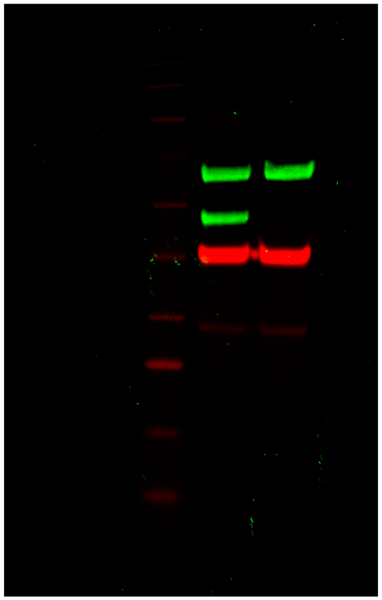

2575-1-AP
1:300

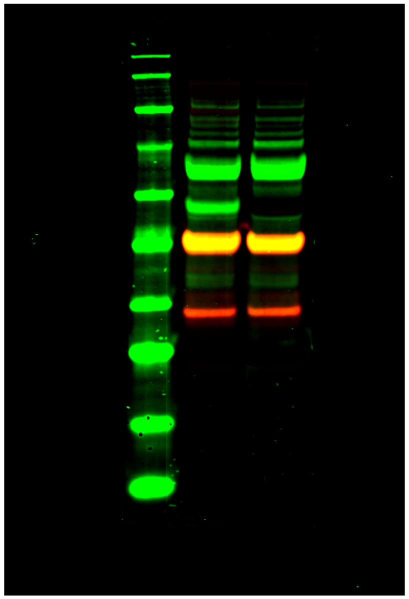

B01-5F2
1:1000

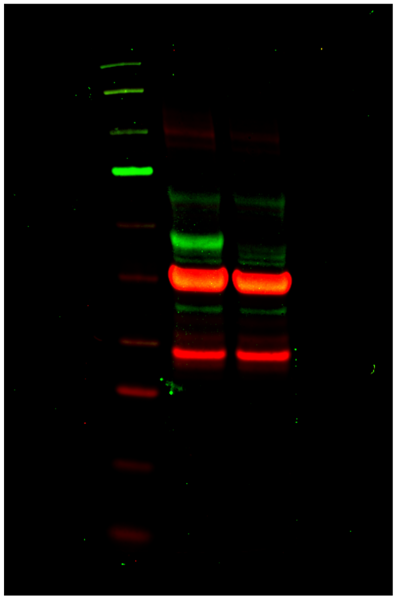

64196
1:1000

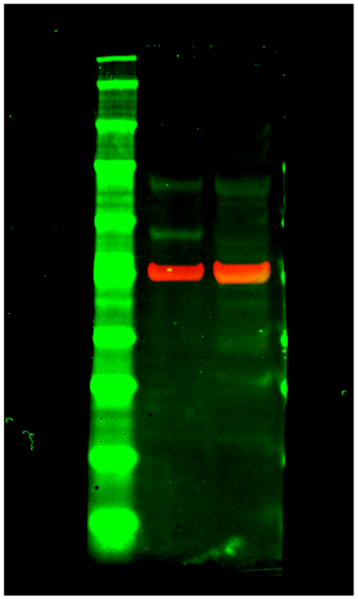

MBS767381
1:1000

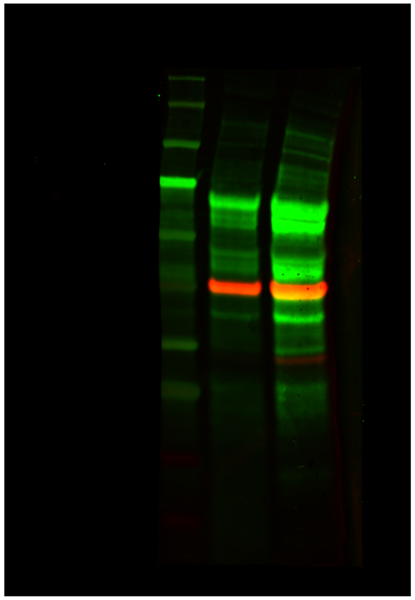

MABN2310
1:1000

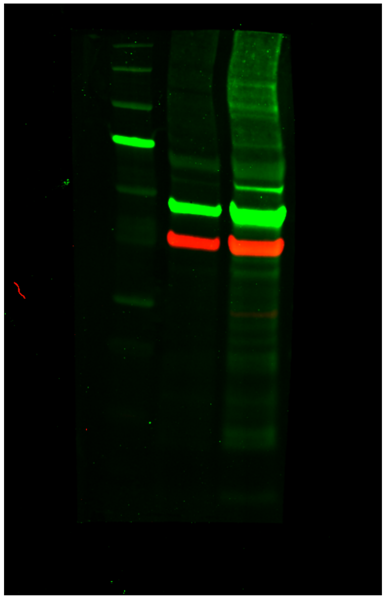

MABN2296
1:250

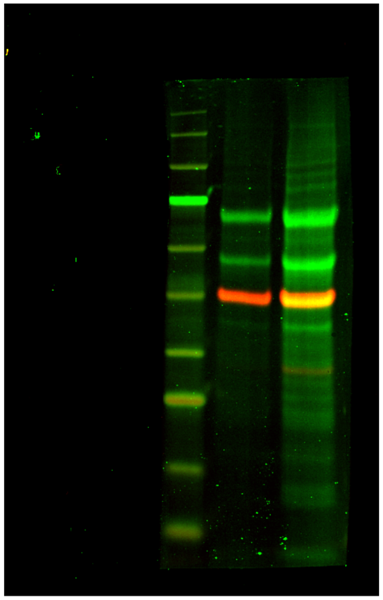

66140-1-IG
1:500

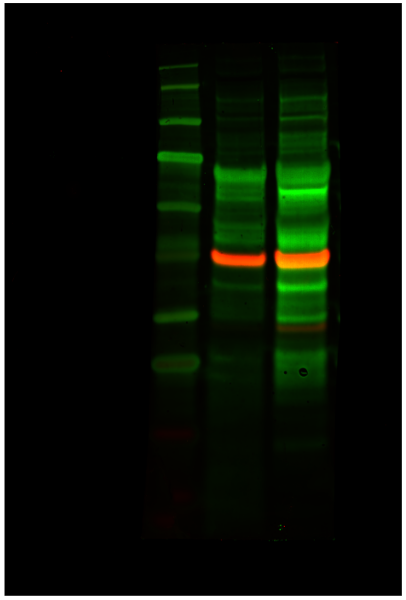

Supplemental Figure 4. Uncropped Western blot images from Figure 5 (main text)**.**

ddPCR Design	Probe target	Primer sequence or MIQE	Probe sequence	Fluorophore
1A-long transcript (variant 3)	Exon 1A-exon 2 junction (NM_001256054.2)	F primer: tgcccggttgcttctcttt<br>R primer: tcttggcaacagctggagatg	GTTTAGGAGATATCTCCGGAGCATT	FAM
1B transcript (variant 2)	Exon 1B-exon 2 junction (NM_018325.4)	F primer: CGGTGGCGAGTGGATATCTC<br>R primer: TGGGCAAAGAGTCGACATCA	TAATGTGACAGTTGGAATGC	FAM
Exon 2-3 (total sense mRNA)	Exon 2-3 junction (total mRNA)	F primer: acctcccacttcatagagtgtgtgt<br>R primer: accctgatcttccattctctctgtgc	AGAATATGGATGCATAAGGAAAGAC	HEX
UBE2D2 HEX	Housekeeping gene (NM_003339, NM_181838)	F primer: ggtctccagcactaactatttcaaaa<br>R primer: caggcactaaaggatcatctggat	ttgtccatctgttctctgttgtgtgatccc	HEX
UBE2D2 FAM	Housekeeping gene NM_003339, NM_181838	F primer: ggtctccagcactaactatttcaaaa<br>R primer: caggcactaaaggatcatctggat	ttgtccatctgttctctgttgtgtgatccc	FAM

Supplemental Table 1. ddPCR primers and probes used to generate the data reported in Figure 4 (main text).

Western blot antibodies					
**Primary antibody (Cat #)**	**Manufacturer/Supplier**	**Target**	**Primary antibody dilution**	**Secondary antibody**	**Secondary antibody dilution**
GTX632041	GeneTex	C9orf72	1:100	Licor IRDye 800CW Donkey anti-mouse	1:1000
GTX634482	GeneTex		1:100		
MBS767381	MyBioSource		1:1000		
66140-1-IG	Proteintech		1:500		
B01-5F2	Bio-Rad		1:1000		
25757-1-AP	Proteintech		1:300	Licor IRDye 680RD Donkey anti-rabbit	
64196	Cell Signaling		1:1000		
MABN2310	Millipore		1:1000	Licor IRDye 800CW Donkey anti-rat	
MABN2296	Millipore		1:250		
MAB8929	R&D Systems	Beta-actin	1:1000	Licor IRDye 800CW Donkey anti-mouse	
NB600-532	Novus Biologicals		1:1000	Licor IRDye 680RD Donkey anti-rabbit	

Immunocytochemistry antibodies					
**Primary antibody (Cat #)**	**Manufacturer/Supplier**	**Target**	**Primary antibody dilution**	**Secondary antibody**	**Secondary antibody dilution**
GTX632041	GeneTex	C9orf72	1:100	Goat anti-mouse IgG (H+L) Alexa Fluor 488	1:500
GTX634482	GeneTex		1:100		
66140-1-IG	Proteintech		1:500		
MBS767381	MyBioSource		1:50		
22637-1-AP	Proteintech		1:500	Goat anti-rabbit IgG (H+L) Alexa Fluor 488	
25757-1-AP	Proteintech		1:300		
ABN1645	Millipore		1:500		
MABN2310	Millipore		1:500	Goat anti-rat IgG (H+L) Alexa Fluor 488	
CL488-66140	Proteintech		1:50	NA	NA

MSD immunoassay antibodies				
**Primary antibody (Cat #)**	**Manufacturer/Supplier**	**Target**	**Capture antibody dilution**	**Detection antibody dilution**
MABN889	Millipore	Poly-GA	1 µg/mL	2 µg/mL
TALS828.179 (lot A-I 0756)	TargetALS	Poly-GP	2 µg/mL	4 µg/mL
TALS828.179 (lot A-I 0757)	TargetALS	Poly-GP		
24494-1-AP	Proteintech	Poly-GP		
23978-1-AP	Proteintech	Poly-GR		
MABN778	Millipore	Poly-GR		
ABN1356	Millipore	Poly-PA		
MABN1790	Millipore	Poly-PA		
23979-1-AP	Proteintech	Poly-PR		
ABN1354	Millipore	Poly-PR		

Supplemental Table 2. Commercially available antibodies tested in experiments reported in Figures 5-6 (main text) and S2.

Human tissue						
**Genotype**	**Brain region**	**Sex**	**Age (years)**	**Diagnosis**	**PMI (hours)**	**Provided by**
C9orf72 repeat expansion	Cerebellum	Male	70	ALS	6	John Ravits, University of California, San Diego
No *C9orf72* mutation	Cerebellum	Male	75	None	8	Brent Harris, Georgetown University

Mouse tissue						
**Genotype**	**Brain region**	**Sex**	**Age (weeks)**	**Jax Labs ID**	**Background**	**Collected by**
C9orf72 500-repeat BAC transgenic	Striatum	Female	22	JAX 029099	FVB/NJ	Victor van Laar, The Ohio State University
Wild-type	Striatum	Female	22	JAX 029099	FVB/NJ	Victor van Laar, The Ohio State University

Supplemental Table 3. Human brain donor and mouse model information for tissues used in assays reported in Figure 6 (main text).

	Poly-GA (MABN889)			Poly-GP (A-I 0756)			Poly-GP (A-I 0757)			Poly-GP (24494-1-AP)			Poly-GR (23978-1-AP)		
**Sample**	Signal	Average of duplicates	Ratio over ctrl	Signal	Average of duplicates	Ratio over ctrl	Signal	Average of duplicates	Ratio over ctrl	Signal	Average of duplicates	Ratio over ctrl	Signal	Average of duplicates	Ratio over ctrl
C9 ALS/FTD cerebellum	1325	1293.5	21.4	3634	3535	7.3	7483	7626.5	7.6	16858	16634.5	1.6	2441	2413.5	0.5
	1262			3436			7770			16411			2386		
Control human cerebellum	64	60.5		484	483.5		1026	1002		11784	10612.5		5452	5351	
	57			483			978			9441			5250		
C9 BAC mouse striatum	344	367.5	8.1	308	318	1.9	757	739	1.8	12478	11544	1.0	3592	2548.5	0.9
	391			328			721			10610			1505		
Wild-type mouse striatum	46	45.5		165	164.5		413	419		11033	11434.5		2996	2962	
	45			164			425			11836			2928		
C9 ALS/FTD iPSC-MNs	186	186.5	3.1	206	203.5	1.6	505	502	1.8	14785	13822.5	1.1	3930	4008.5	0.8
	187			201			499			12860			4087		
C9orf72 KO iPSC-MNs	62	61		121	124		286	286.5		12835	13126.5		4967	4822	
	60			127			287			13418			4677		

	Poly-GR (MABN778)			Poly-PA (ABN1356)			Poly-PA (MABN1790)			Poly-PR (23979-1-AP)			Poly-PR (ABN1354)		
**Sample**	Signal	Average of duplicates	Ratio over ctrl	Signal	Average of duplicates	Ratio over ctrl	Signal	Average of duplicates	Ratio over ctrl	Signal	Average of duplicates	Ratio over ctrl	Signal	Average of duplicates	Ratio over ctrl
C9 ALS/FTD cerebellum	68	71.5	1.0	196	190.5	1.1	1603	1657.5	0.5	11326	11612.5	1.0	574	562.5	1.1
	75			185			1712			11899			551		
Control human cerebellum	70	69		169	168.5		3010	3014.5		11637	11209		506	519	
	68			168			3019			10781			532		
C9 BAC mouse striatum	71	73	1.1	220	218.5	1.2	6087	6066.5	0.8	12027	11970	1.0	1525	1520.5	2.4
	75			217			6046			11913			1516		
Wild-type mouse striatum	64	66.5		180	183.5		6981	7373		12112	12180		641	633.5	
	69			187			7765			12248			626		
C9 ALS/FTD iPSC-MNs	61	66	0.9	217	212.5	1.1	953	955.5	0.4	12375	12158	0.9	691	759	1.0
	71			208			958			11941			827		
C9orf72 KO iPSC-MNs	73	73		186	189.5		2186	2205.5		13227	12972		822	757.5	
	73			193			2225			12717			693		

Supplemental Table 4. Electrochemiluminescence signals from 10 commercial dipeptide repeat protein antibodies tested in three sample types on MSD immunoassay.

			spCas9 gRNA		Excision Primers			5' Cut Site Primers			3' Cut Site Primers		
Edit Name	Edit	Excision Size	5' gRNA	3' gRNA	F primer	R primer	Expected excision band amplicon length (bp)	F primer	R primer	Expected 5' cut site amplicon length (bp)	F primer	R primer	Expected 3' cut site amplicon length (bp)
**Repeat expansion excision**	repeat expansion excision	7 NT if no RE, 607 NT if 200 repeats	AACTCAGGAGTCGCGCGCTA	ggcccgccccgaccacgccc	CCGCTAGGAAAGAGAGGTGCG	GAGGAGAGCCCCCGCTTCTAC	505 (unedited WT allele), unedited RE will not amplify, 480 (edited)	N/A	N/A	N/A	N/A	N/A	N/A
**1Ax**	exon 1A excision	227bp	TGCGATGACGTTTTCTCACG	TACTGTGAGAGCAAGTAGTG	GATCCAGCAGCCTCCCCTAT	GCTACAGGCTGCGGTTGTTT	440 (unedited), 214 (edited)	TCCAGCAGCCTCCCCTATT	TTTACGTGGGCGGAACTTGT	216	AGAGAGGTGCGTCAAACAGC	CTCCTGAGTTCCAGAGCTTGC	282
**1Bx**	exon 1B excision	124bo	CGTGGTCGGGGCGGGCCCGG	GCTGTTTGGGGTTCGGCTGC	TACTCGCTGAGGGTGAACAAG	CAGTCGCTAGAGGCGAAAGC	390 (unedited), 266 (edited)	N/A	N/A	N/A	GTGGCTGTTTGGGGTTCGG	CCAGTCGCTAGAGGCGAAAG	137
**KO (WT line)**	Excision from 5' to exon 1A through exon 2	7,205bp	TGTGCGAACCTTAATAGGGG	AATGGGGATCGCAGCACATA	GCAGACCAAAAGACGCAAGG	ACCAGAAAATAAGCTTTCAACAGAT	Unedited will not amplify (7748), 578 (edited)	GCAGACCAAAAGACGCAAGG	CAGCGAGTACTGTGAGAGCA	751	GGGTTAGGGGCCAAATCTCC	ACCAGAAAATAAGCTTTCAACAGAT	932
**KO (patient line)**	Excision from 5' to exon 1A to exon 3-4 intron (using all 4 allele-specific gRNAs in 1 reaction)	21kb	CTCTGTGAGAAGTTTTTATC and CTCTGTGAGAAGTTCTTATC	GACTTAGAAGAAATATTGTG and GACTTAAAAGAAATATTGTG	AGGAACCAAGCAGCCATGAA	GGGAAGCCACACCCTTGTAA	Unedited will not amplify (21436), 392 (edited)	CTTTGGCACAGATAGGCCAC	GGCAGGGTGACTGCTTTAAC	199	TGCCCAGAATAAATTTTGGATAACT	GGGAAGCCACACCCTTGTAA	337

Supplemental Table 5. **Guide RNAs and primers used to generate and verify, respectively, each edited cell line.** We used spCas9 with a protospacer adjacent motif (PAM) of NGG (not included in the gRNA sequence). Excision size is provided for each type of excision in the WT line. Expected amplicon size for each set of PCR primers is also provided.
